# The association between the well-being and workability among university staff during the COVID‑19 pandemic

**DOI:** 10.1192/j.eurpsy.2023.1677

**Published:** 2023-07-19

**Authors:** I. Sellami, A. Abbes, A. Haddar, N. Kotti, M. L. Masmoudi, K. Jmal Hammami, M. Hajjaji

**Affiliations:** 1 occupational medecine, Hedi Chaker Hospital, University of Sfax; 2 occupational medecine, University of Sfax; 3 Hedi Chaker Hospital, University of Sfax, Sfax, Tunisia

## Abstract

**Introduction:**

The coronavirus disease 2019 (COVID-19) pandemic has had an unprecedented impact on educational systems and the well-being of the university staff. The mental health of university staff can affect their ability to work.

**Objectives:**

Our study aimed to assess the association between the well-being of university staff and their workability during the COVID-19 pandemic.

**Methods:**

We conducted a cross-sectional study among university staff in Sfax, Tunisia. We collected data between September and October 2021 using a self-administered questionnaire including socio-professional characteristics, the Work Ability Score (WAS) and the Arabic version of the Mental Health Continuum-Short form.

**Results:**

Our sample was composed of 62 university staff. The respondents’ mean age was 51.4±6.7 years and 67.7% were female. The average job tenure was 17.9 ± 8 years. The mean score of WAS was 7.5±1.8. The mean scores of emotional well-being, social well-being and psychological well-being were 8.5±4.2, 12.6±6.1, and 19.9±7.3, respectively. Sixty-one per cent of participants reported languishing to moderate mental health, and 39% % were flourishing. The workability of participants was significantly associated with their well-being (p = 0.04).

**Conclusions:**

Having good mental health improves the worker’s ability to work. Taking care of mental health is crucial to guarantee better efficacy at work.

**Disclosure of Interest:**

None Declared

